# Supplementary figures and images for: Estrogen receptor—positive breast cancer survival prediction and analysis of resistance–related genes introduction
Source: PeerJ. 2021 Oct 26;9:e12202. doi: 10.7717/peerj.12202 (PMC8555508; doi:10.7717/peerj.12202)

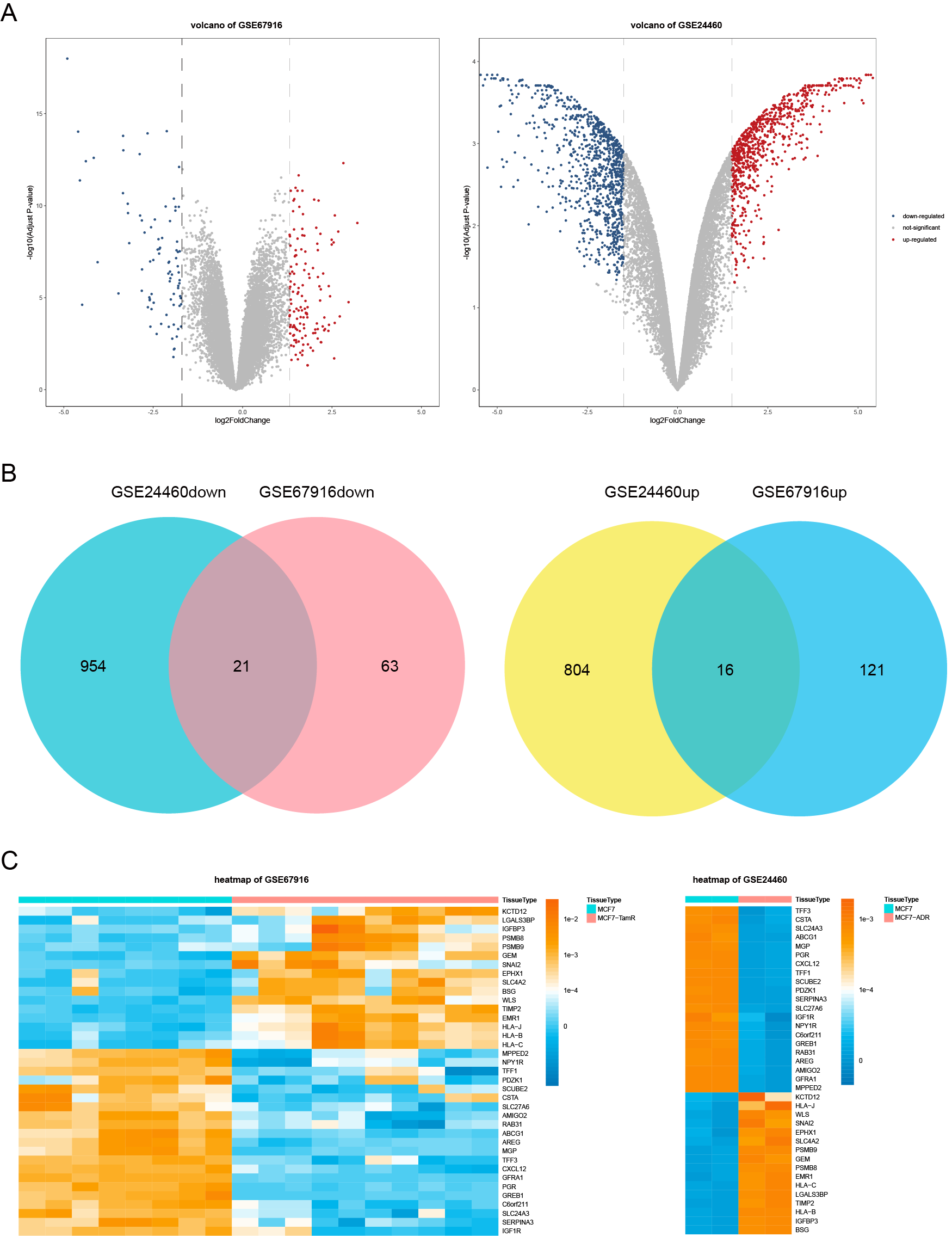

Supplement: Supplemental Information 1 — (A, B) The volcano plots of the DEGs in GSE67919 and GSE24460. (C, D) The genes both up-regulated and down-regulated in GSE67916 and GSE24460, respectively. (E, F) The heatmap of the DEGs in GSE67919 and GSE24460. [file peerj-09-12202-s001.png]

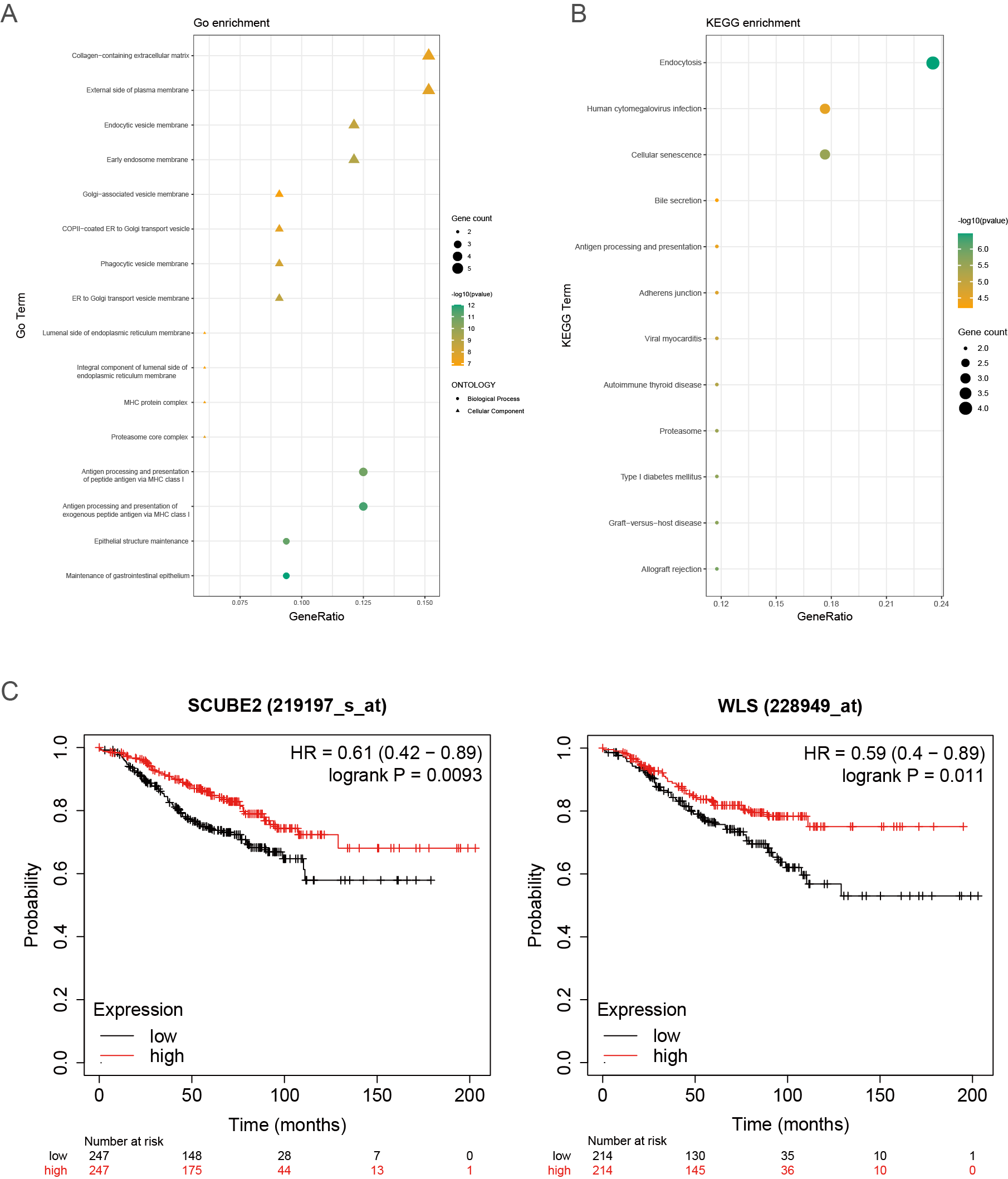

Supplement: Supplemental Information 2 — (A, B) Representative results of GO and KEGG analyses. (C, D) The prognosis of SCUBE2 (C) and WLS (D) in the Kaplan–Meier Plotter website. [file peerj-09-12202-s002.png]

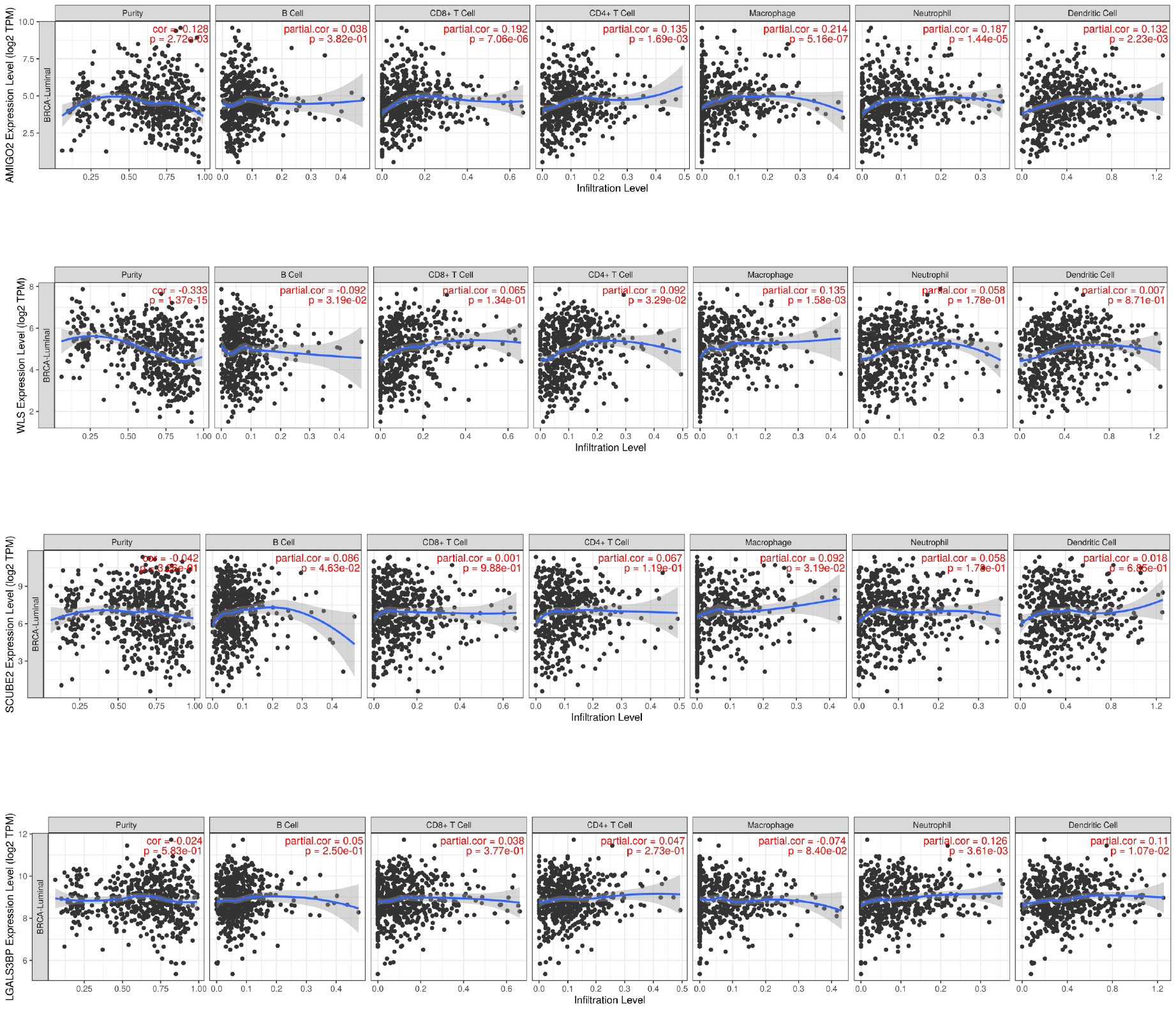

Supplement: Supplemental Information 3 [file peerj-09-12202-s003.png]
